# Apparent Death—Premature Interment

**Published:** 1848-07

**Authors:** 


ACADEMY OF SCIENCES.

*Apparent Death. Premature Interments.*—This question was made the subject of a competition for a prize, to be awarded in 1848. M. Rayer reported on the comparative value of the essays presented to the Academy.

The two following questions had been proposed by the Academy:—1. What are the characters of apparent death? 2. By what means can premature interments be prevented? Amongst the numerous memoirs presented on this subject, one only, that of Dr. Bouchut, had appeared to the commission to deserve a recompense. According to Dr. Bouchut, the certain signs of death were immediate, or occurred only after a certain time. The former were three in number, viz., the prolonged cessation of the pulsations of the heart; the simultaneous relaxation of all the sphincters, due to the paralysis of these muscles; and the collapse of the eyeball, together with the dimness of the cornea. In the opinion of the commission these signs had not all an equal value: from observations made on the human subject, and experiments on animals performed by the commission and by Dr. Bouchut, the reporter thought himself justified in asserting that the absence of pulsation of the heart, ascertained by auscultation during a period of five minutes, could leave no doubt on the cessation of life. This sign of death appeared to the commission to derive an additional degree of certainty from the fact that the definitive arrest of the movements of the heart caused immediate cessation of respiration and of the accomplishment of the functions of the nervous system, when it was not preceded by them. The simultaneous relaxation of the sphincters and the collapse of the eyeball did not, said the reporter, present the same degree of certainty with regard to the reality of death.

As to the subsequent and certain signs of death, M. Bouchut also admitted three, viz., rigidity, absence of muscular contraction under the influence of galvanic stimulus, and putrefaction; the certainty of these signs was admitted by all professors of forensic medicine, and was incontrovertible.

In conclusion, the reporter observed that four positive signs of death existed: prolonged cessation of the sounds of the heart, rigidity, absence of contractility under galvanic stimulants, and general putrefaction. The three former occurring long before the last-mentioned sign, it was not necessary to await the latter in order to proceed to the operations of embalming and inhumation, but that the recognition of the three former signs should be left to the experience of a physician; and finally, that as it was possible to ascertain positively the reality of death without waiting for the putrefaction of the subject, the establishment of dead-houses on the German plan was unnecessary. Dr. Bouchut's memoir was the best which had been presented on these questions for ten years, and the commission proposed that the prize be awarded to him.—*London Medical Times.*

*Sale of Horse-flesh*—Since the siege of Copenhagan in 1807, horse-flesh has been regularly sold by the butchers in that capital for general consumption. The only formality required is, that a horse, before it is killed, should be examined by a veterinary surgeon, and marked on each hoof. There are even shambles especially licensed, where nothing but horse-flesh is sold. The establishment is placed under the immediate superintendence of the Veterinary College. In Belgium, the horse-flesh obtained from Meulenbeck-Saint-Jean, where a large number of horses are killed, is publicly sold, and when it gets stale is used for sausages. We are afraid that the British poor consume unconsciously a good deal of the same article.—*Lancet.*

